# Is a Small-Pox Hospital a Nuisance?

**Published:** 1895-12-14

**Authors:** 


### The Hospital

AN INSTITUTIONAL JOURNAL OF

**The Medical Sciences and Hospital Administration,**

WITH

**AN EXTRA NURSING SUPPLEMENT.**

VOL. XIX.—No. 481.] [DEC. 14, 1895.

### IS A SMALL-POX HOSPITAL A NUISANCE?

By a decision given in the High Court of Justice, Chancery Division, on November 20th, a matter was decided which has for years been a puzzle to sanitary authorities. Everyone is agreed as to the desirability of providing special hospitals for the isolation of small-pox, but no one seems ready to admit a small-pox hospital into his neighbourhood. Hence endless difficulties. The Guildford, Godalming, and Woking Joint Hospital Board desired to erect a small-pox hospital for the use of their district, and for that purpose chose a plot of land a little over two acres in extent and about a hundred yards across. The usual objections were raised, and an action was brought to restrain them from so using it. The land was surrounded on all sides by common, on which it was proved that cattle and geese were fed and children played; and among the plaintiffs were persons having houses 60, 110, 130, 300, and 33[illegible] ards from the boundary. Among the witnesses called to support the proposition that the p[illegible]oposed hospital would be a nuisance were Dr. Thorne Thorne, and other officials from the Local Government Board, who gave evidence as to the danger of infection from the proximity of a small-pox hospital. Full emphasis was thus given to the official view that small-pox could in some unexplained manner be conveyed for long distances by "aerial convection," Dr. Thorne Thorne stating that he was convinced that the distance to which the infection might be carried through the atmosphere extended to at least a mile. Evidence, however, was given on the other side showing that groups of persons living in close proximity to small-pox hospitals had not suffered. Mr. Justice Kekewich, in giving judgment, entirely put on one side all the points that had been raised as to whether other sites were not more suitable; all he had to consider was whether the use of this cottage, or the use of this land, for the purposes of a small-pox hospital gave rise to "a real apprehension of a real danger, not a sentimental or fanciful one."

In regard to this he pointed out the great difference there was between the treatment of small-pox in such a cottage as now existed and its treatment in a proper hospital. It had been proved that this cottage, as it stood, was not a proper place for the reception of patients, but the evidence went to show that there would be no difficulty in erecting on this plot a building capable of accommodating ten or twelve patients and providing everything necessary, according to the exigencies of modern science, for the attendance on those patients. A great deal depended upon administration, and if the hospital were not properly administered there might be a good chance of success in an action to prevent the continuation of the hospital on the suggested terms. The further question was, "Is aerial convection a theory and hypothesis sufficiently established to enable the Court judicially to say this constitutes a source of danger against which the defendants have to provide?" If it was ever proved scientifically and beyond all reasonable doubt that infection could be conveyed possibly a quarter of a mile, half a mile, or even a mile, in spite of brick walls, doors, and windows, it might be a question whether something should not be done to deprive the public of the blessing arising from such a hospital as this, even though some persons might in that case suffer. But at present his Lordship was not satisfied that this had been established. What one medical witness had called "particulate matter" no one had seen and no one had analysed. Whether it went half a mile or a mile, or how far it went, it was impossible to say. It was not traceable. Under such circumstances it would be entirely wrong to grant an injunction against the defendants erecting this hospital because some learned men whose opinions entitled them to great respect came to the conclusion, though they could not prove it, that small-pox was the subject of aerial convection. His Lordship found no reported case to assist him in coming to a conclusion at all; the case came before him almost as *res integra*. On the grounds given he was of opinion that he should be going much too far in saying that there was any real kind of apprehension of a real danger; and, that being so, the action so far failed.

The importance of this decision cannot be exaggerated, setting sanitary authorities free, as it does, from the incubus which has been laid on them so long by this official theory of "aerial convection." At the same time sanitary authorities must bear in mind that this decision by no means leaves them free to utilise as a small-pox hospital any hovel which may be available. The plaintiffs definitely won their case in preventing the conjoint board from using the existing cottage. A small-pox hospital must be properly constructed and provided with proper appliances, and even then will be subject to legal process if it be proved to be a nuisance. But the nuisance must be proved, and the "bogey" of "aerial conv ction" of infection can no longer be raised against it.

